# Supplementary material for: EnzML: multi-label prediction of enzyme classes using InterPro signatures
Source: BMC Bioinformatics. 2012 Apr 25;13:61. doi: 10.1186/1471-2105-13-61 (PMC3483700; doi:10.1186/1471-2105-13-61)
Supplement: Addtional file 5 — The Java code to format the data files, evaluate and predict. The file enzml_java_code.tar.gz contains the Java code used to format database data to ARFF and XML formats, to execute cross and train-test (jackknife) evaluations and to record evaluation results to database. More information is included in the readme.txt file and the Javadoc files. The code can be used with a MySQL database. To use a different database software, other JDBC drivers might be required. [file 1471-2105-13-61-S5.gz › java_code/utils/doc/uk/ac/ed/inf/utils/tests/OneToManyMapTest.html]

OneToManyMapTest


---


|  |  |  |
| --- | --- | --- |
| **Overview** \| **Package** \| **Class** \| **Use** \| **Tree** \| **Deprecated** \| **Index** \| **Help** | |  |
| **PREV CLASS**   **NEXT CLASS** | **FRAMES**    **NO FRAMES**     **All Classes** |  |
| SUMMARY: NESTED \| FIELD \| CONSTR \| METHOD | DETAIL: FIELD \| CONSTR \| METHOD |  |


---


## uk.ac.ed.inf.utils.tests Class OneToManyMapTest

```
java.lang.Object
  junit.framework.Assert
      junit.framework.TestCase
          uk.ac.ed.inf.utils.tests.OneToManyMapTest
```

**All Implemented Interfaces:**: junit.framework.Test

---

``` public class OneToManyMapTest extends junit.framework.TestCase ```

Class

**Version:**
:   3 Jun 2008

**Author:**
:   Luna De Ferrari luna.deferrari-at-ed.ac.uk

---

**Constructor Summary**

`OneToManyMapTest()`


| **Method Summary** | |
| --- | --- |
| `static OneToManyMap` | `oneToManyMap1()` |
| `static OneToManyMap` | `oneToManyMap2()` |
| `void` | `testAddAll()` |
| `void` | `testAddKeyValue()` |
| `void` | `testGetKeysVectorValuesVector()` |
| `void` | `testGetValuesForAKey()` |
| `void` | `testMapIsFilled()` |
| `void` | `testRemoveKeyValue()` |

**Methods inherited from class junit.framework.TestCase**

`countTestCases, getName, run, run, runBare, setName, toString`

**Methods inherited from class junit.framework.Assert**

`assertEquals, assertEquals, assertEquals, assertEquals, assertEquals, assertEquals, assertEquals, assertEquals, assertEquals, assertEquals, assertEquals, assertEquals, assertEquals, assertEquals, assertEquals, assertEquals, assertEquals, assertEquals, assertEquals, assertEquals, assertFalse, assertFalse, assertNotNull, assertNotNull, assertNotSame, assertNotSame, assertNull, assertNull, assertSame, assertSame, assertTrue, assertTrue, fail, fail, failNotEquals, failNotSame, failSame, format`

**Methods inherited from class java.lang.Object**

`equals, getClass, hashCode, notify, notifyAll, wait, wait, wait`

**Constructor Detail**

### OneToManyMapTest

```
public OneToManyMapTest()
```


**Method Detail**

### testAddAll

```
public void testAddAll()
```

---


### testAddKeyValue

```
public void testAddKeyValue()
```

---


### testGetKeysVectorValuesVector

```
public void testGetKeysVectorValuesVector()
```

---


### testGetValuesForAKey

```
public void testGetValuesForAKey()
```

---


### testMapIsFilled

```
public void testMapIsFilled()
```

---


### testRemoveKeyValue

```
public void testRemoveKeyValue()
```

---


### oneToManyMap1

```
public static OneToManyMap oneToManyMap1()
```

---


### oneToManyMap2

```
public static OneToManyMap oneToManyMap2()
```


---


|  |  |  |
| --- | --- | --- |
| **Overview** \| **Package** \| **Class** \| **Use** \| **Tree** \| **Deprecated** \| **Index** \| **Help** | |  |
| **PREV CLASS**   **NEXT CLASS** | **FRAMES**    **NO FRAMES**     **All Classes** |  |
| SUMMARY: NESTED \| FIELD \| CONSTR \| METHOD | DETAIL: FIELD \| CONSTR \| METHOD |  |


---
